# Parent-identified barriers to accessing exposure therapy: A qualitative study using process mapping

**DOI:** 10.3389/fpsyt.2023.1068255

**Published:** 2023-03-20

**Authors:** Hannah E. Frank, Grace Cain, Jennifer Freeman, Kristen G. Benito, Erin O’Connor, Josh Kemp, Bo Kim

**Affiliations:** ^1^The Warren Alpert Medical School of Brown University, Providence, RI, United States; ^2^Bradley Hospital, East Providence, RI, United States; ^3^VA Center for Healthcare Organization and Implementation Research, Boston, MA, United States; ^4^Harvard Medical School, Boston, MA, United States

**Keywords:** exposure therapy, anxiety, obsessive-compulsive disorder, dissemination, treatment access, qualitative, parents

## Abstract

**Background:**

Youth with anxiety and obsessive–compulsive disorder (OCD) rarely access exposure therapy, an evidence-based treatment. Known barriers include transportation, waitlists, and provider availability. Efforts to improve access to exposure require an understanding of the process that families take to find therapists, yet no prior studies have examined parents’ perspectives of the steps involved.

**Methods:**

Parents of children who have received exposure therapy for anxiety and/or OCD (*N = 23*) were recruited from a hospital-based specialty anxiety clinic where the majority of their children previously received exposure. Recruitment was ongoing until thematic saturation was reached. Parents completed questionnaires and attended an online focus group during which they were asked to describe each step they took—from recognizing their child needed treatment to beginning exposure. A process map was created and shown in real-time, edited for clarity, and emailed to parents for member checking. Authors analyzed process maps to identify common themes.

**Results:**

Several themes emerged, as visually represented in a final process map. Participants identified a “search-outreach” loop, in which they repeated the cycle of looking for therapists, contacting them, and being unable to schedule an appointment due to factors such as cost, waitlists, and travel time. Parents often did not know about exposure and reported feeling guilty about their lack of knowledge and inability to find a suitable provider. Parents reported frustration that medical providers did not often know about exposure and sometimes dismissed parents’ concerns. Participants emphasized the difficulty of navigating the mental health system; many reported that it took years to find an exposure therapist, and that the search was sometimes stalled due to fluctuating symptoms.

**Conclusion:**

A common thread among identified barriers was the amount of burden placed on parents to find treatment with limited support, and the resultant feelings of isolation and guilt. Findings point to several directions for future research, such as the development of parent support groups for navigating the mental health system; enhancing coordination of care between medical and mental health providers; and streamlining referral processes.

## Introduction

Youth with mental health problems have limited access to evidence-based interventions (EBIs). Although nearly 14% of youth have a mental health disorder (1), a minority of adolescents with these disorders access mental health treatment (2). Furthermore, adolescents with internalizing symptoms access treatment at lower rates than those with externalizing symptoms (2), highlighting how the treatment access crisis particularly affects anxious and depressed youth. Treatment access refers to the ability “to identify healthcare needs, to seek healthcare services, to reach, to obtain or use health care services, and to actually have the need for services fulfilled” [pg., 8, (3)]. A variety of factors may impact access to care, such as lack of transportation, long waitlists, and few available providers trained in EBIs (4). Factors such as inaccurate diagnosis of symptoms (5), limited consumer knowledge about EBIs (6), and minimal provider adoption of EBIs (7–9) all contribute to the difficulties of accessing appropriate care.

Exposure therapy for anxiety disorders and obsessive–compulsive disorder (OCD) is a particular striking example of an intervention that has strong empirical support (10) but is rarely used in routine clinical care settings (11), making it challenging for families to access. Although many efforts have been made to increase provider use of exposure therapy [e.g., through training providers (12)], it is still unlikely that an individual with an anxiety disorder or OCD will be able to access exposure therapy (13) or that it will be delivered effectively in a routine clinical care setting (14). Without adequate access to effective mental health care, anxious youth continue to struggle with symptoms that often persist into adulthood (13, 15, 16). Further research is needed to address the barriers parents and youth face when seeking exposure therapy for anxiety disorders and OCD.

The field of implementation science provides guidance for addressing barriers to accessing EBIs. Implementation science is the study of how to translate research findings into routine clinical practice settings, with the goal of improving the quality of services (17). Implementation science offers a wide array of conceptual frameworks that identify barriers and facilitators, or determinants, of EBI uptake across different domains and stages of implementation (18, 19). Assessment of these determinants is well documented for a variety of EBIs. The goal of identifying determinants is to guide the selection of implementation strategies that will increase the use and availability of EBIs (20). However, the process of selecting implementation strategies and tailoring them to a unique context and population is often a difficult and ill-defined task (21, 22). When researchers spend insufficient time understanding the barriers of a context or apply implementation interventions without matching them to the barriers they seek to mitigate, implementation efforts may yield ineffective results. In other words, implementation frameworks guide the selection of strategies that may improve the process of accessing EBIs, but such framework-guided strategy selection cannot be optimized without exactly “locating” where barriers are within the process and how they vary across contexts. Although existing research has gained insight from community partners to address this concern, no existing studies have focused on understanding parents’ perspectives of determinants to accessing EBIs for anxiety disorders and OCD.

Parents and caregivers are vital players in accessing treatment for anxious youth and can provide researchers with a direct view into the barriers and facilitators families face during the treatment seeking process. For youth with anxiety and OCD, seeking mental health services is often primarily driven by parents (23–25) and research suggests that adolescent treatment seeking is influenced by others, with the strongest influence coming from parents (26). Parents who have accessed EBIs for their children may be particularly well-positioned to identify possible solutions to address barriers to treatment access given their intimate knowledge of barriers faced during their own search. Past research indicates that parents identify determinants to accessing treatment for their children in a variety of categories. A systematic review found that families identified barriers in the form of: (1) structural barriers (e.g., wait times, cost); (2) individual-level barriers including (a) family attitudes towards treatment; (b) limited family knowledge of mental health problems and how to seek and access help; and (c) family circumstances such as a family’s support network (27). In another study that asked parents about barriers to seeking outpatient services for their children, 60.3% reported lack of information about where to seek help as a barrier, 59.8% reported professionals not listening as a barrier, and 53.7% reported providers not initiating treatment or issuing referrals as a barrier (28). While past research highlights the depth of knowledge that parents hold regarding their experience seeking services for their children, no prior studies have examined the unique process families go through to access EBIs for anxiety and OCD, including the timeline and barriers involved in accessing treatment.

Process mapping (29) is one method that can be used to systematically locate determinants to treatment access and guide optimal implementation strategy selection. Process mapping, which was originally developed and applied within the fields of business and engineering, is widely used for quality improvement in health and medical settings, and it has begun to be adapted for implementation efforts (30, 31). A process map is a detailed flow chart that makes work processes visible and identifies each of the actors and their roles in a process (29). Process mapping is a data-driven approach that identifies the steps in complex, multi-step activities and allows for assessment of inefficiencies and the development of more appropriate and effective systems (31–33). Given that few tools have been successful at assessing context prior to implementation or sustainability efforts (34), process mapping may be helpful to improve the selection of strategies that are uniquely appropriate for the local context, which will in turn increase the availability of EBIs and improve access to effective treatments. Process mapping provides a method for parents to identify the distinctive determinants they faced in their process of searching for services, and for the synthesis of parent experiences to identify common “stuck-points” in the process across individuals.

This study used process mapping to develop an in-depth understanding of parents’ efforts to access exposure therapy for their children, including inefficiencies and barriers encountered in the process. Developing a process map of accessing exposure therapy for anxiety is especially important given that: (1) there is robust evidence for the efficacy of exposure therapy, yet (2) significant barriers to accessing it persist. Although the barriers and facilitators to using exposure are well-documented (35), there is still disconnect between understanding barriers and identifying appropriate and effective implementation strategies to address these barriers. Process mapping may be well-suited to unpack this “black box” and understand the types of implementation strategies most appropriate to a specific context. Thus, the aim of this study was to use process mapping to identify specific barriers that parents face when trying to access exposure therapy, as well as to identify potential parent-identified solutions to address them.

## Methods

### Participants

Participants (*N* = 23) included parents of children (and one former patient) with anxiety and/or OCD who have received exposure therapy in any setting (i.e., community mental health, outpatient hospital-based clinic, and partial hospital program). Four participants were recruited from an existing parent advisory group, 12 from an outpatient hospital-based clinic study, one from a partial hospital program, and five from unknown sources. Other inclusion criteria included English-speaking and willingness to complete study procedures. There were no exclusion criteria.

### Measures

#### Demographics questionnaire

Participants completed a questionnaire assessing parent and child demographic characteristics (e.g., age, sex, gender, race, and ethnicity).

#### Clinical Global Impression Scale-Severity–Parent-rated version

Parents were asked to respond to a modified version of the CGI-S (36) in which they were asked “Please provide a rating for how severe your child’s anxiety/obsessive–compulsive disorder symptoms were at their worst” and “currently.” Responses ranged from 1 (normal, not at all a problem) to 7 (extremely ill), as consistent with the CGI-S.

#### Brief Revised Child Anxiety and Depression Scale

The Revised Child Anxiety and Depression Scale (RCADS-25) is a 25-item parent report measure of anxiety and depressive symptoms (37). Items are rated on a four-point Likert-scale from 0 (*never*) to 3 (*always*). It yields three scores: Total Anxiety, Total Depression, and Total Anxiety and Depression. Parents were asked to rate items based on when their child’s symptoms were at their worst.

### Recruitment and procedures

All study procedures were approved by the Lifespan Institutional Review Board. Data collection tooks place between September 2020 and March 2022.

#### IMPACT advisory group

Prior to formally recruiting participants for this study, we piloted the focus group-based process mapping methods to be used for this study (described below), as part of an existing advisory group meeting for the Improving Access to Child Anxiety Treatment (IMPACT study; *PCORI/IHS-2017C1-6,400*). The IMPACT study is an ongoing comparative effectiveness trial that is comparing different delivery methods for exposure therapy. The IMPACT patient and family advisory group is comprised of 12 participants, all of whom were invited by email to attend an advisory group meeting specifically focused on accessing exposure therapy. Five of the advisory group participants indicated interest, and four attended the focus group. One of these participants was a former (now adult) patient; the remainder were parents. Participants completed the Demographics Questionnaire prior to the focus group, which took place *via* Zoom in September 2020.

#### Accessing exposure study participants

After piloting the process mapping methods with the existing advisory group, we recruited additional participants to attend focus groups and complete online questionnaires as part of the accessing exposure (ACE) study. We used several methods to recruit participants including: (1) sending emails and providing fliers to therapists who provide exposure therapy through the Pediatric Anxiety Research Center (PARC) outpatient clinic and through PARC training studies and asking them to share the study information with patients’ parents who might be interested in participating; (2) posting fliers in clinical space at Bradley Hospital; (3) posting to OCD Rhode Island social media channels; (4) contacting participants who previously completed other PARC studies and who consented to be contacted in the future for other studies conducted through PARC; and (5) asking participants to forward information about the study to parents they knew whose children have also completed exposure therapy.

Parents interested in participating in the study first completed an online study interest form *via* REDCap, a secure, web-based software platform designed to support data collection for research studies (38, 39). Parents then received a link to complete an electronic consent form followed by online questionnaires, which took about 15–30 min to complete. Following completion of quantitative measures, participants were scheduled to attend a focus group *via* Zoom. One family had two parents attend the focus group, and the remainder only had one parent per family attend. After the focus group, participants received payment (a $50 Amazon gift card) by email. Recruitment was ongoing until saturation was reached (i.e., data from additional participants did not provide new information), leading to a sample of 18 ACE study participants across six focus groups between August 2021 and March 2022. Together with the pilot focus group participants, this yielded a final study sample of 23 participants across seven focus groups between September 2020 and March 2022.

#### Focus group (process mapping) procedures and analysis

Focus groups took place online *via* Zoom. Meetings lasted 60–90 min and were audio recorded. Meetings took place with 2–5 parents, except for one parent who met with us individually due to scheduling constraints. Each group was led by a licensed clinical psychologist (HEF) with facilitation assistance from a trained research assistant (GC). In addition, GC took extensive field notes during each group, which were later reviewed as part of the data analytic process. The meeting agenda for each focus group included: (1) introductions of all participants and study team members; (2) a brief overview of the method and purpose of process mapping; (3) a thought exercise during which parents were given 2-min to think about their experiences and the process that they went through to access exposure therapy for their child; and (4) guided discussion of parents’ experience accessing exposure with a simultaneous display of process mapping in real time *via* screen share.

Focus groups followed a structured approach based on process mapping (i.e., collecting information on specific processes related to identifying and scheduling an appointment with an exposure therapist). Parents were told that they would be asked to describe the process from (a) when they made an initial attempt to seek therapy services for their child until; (b) their child began exposure therapy with a trained therapist. Specifically, we inquired about who parents initially contacted when they decided they needed treatment for their child’s anxiety and the process of seeking and following up on referrals to therapists. Parents were encouraged to provide information about what steps they followed at each stage of their search process, including who they talked to and how successful they were in finding appropriate treatment at each step along the way. As parents shared information about the steps they followed, a process map was created and screen shared in real time using Lucidchart, an online software program that allows for easy creation of diagrams and flowcharts (40). The process map displayed the combined experiences of all parents participating in each focus group. During the focus group, parents were asked to confirm whether the process map reflected the information they were sharing, including decision points and problems (e.g., gaps, uncertainties, and bottlenecks) that occurred at each step. After each group, we drafted a refined, electronic version of the process map using Lucidchart that incorporated data from their group and all previous groups (i.e., a *common features process map*). This *common features process map* included elements that were common or similar across families. Saturation was reached when no new process steps were identified. Within 1 week of each focus group, we distributed the *common features process map via* email to participants for their input and confirmation that it accurately represented their family’s process. No parents suggested any changes to the process map in response to these email requests.

#### Data analysis

After the completion of all focus groups, data analysis took place in three steps. Analyses were guided by participants’ experiences and themes that emerged based on what they described during focus groups. First, the first and second author (HEF and GC) reviewed field notes and the *common features process map* to ensure that all key steps of the process were represented. This review of notes was also used to ensure inclusion of all relevant “clouds” (i.e., gaps, bottlenecks, and uncertainties) on the process map. Using information from the *common features process map* and the field notes, HEF and GC created a table that expanded upon “cloud” descriptions by providing examples from the field notes. Using the field notes as a reference point, sections of the focus group audio recordings were reviewed to select representative quotes for each cloud. A visual review of the map alongside the notes also highlighted multiple “loops” that were commonly described by parents in the process of seeking treatment. HEF and GC identified these loops through discussion and consensus.

Second, HEF and GC conducted an inductive content analysis of all field notes to identify whether additional themes emerged that were not reflected in the “clouds” or “loops.” Then, they organized *all* themes, including emergent themes from field notes, as well as themes represented in “clouds” and “loops” *via* Lucidspark, an online software for collaborative ideation and consensus-reaching. This allowed for collaborative grouping of similar themes into larger groups, yielding a total of six overarching themes for the entire dataset.

Third, in June 2022, all participants were sent an updated version of the *common features process map*, as well as a description of the identified “clouds” and loops for member checking. Parents were asked to confirm whether their experience was reflected in the map and whether there was anything that was wrong or missing from the materials. One parent replied with detailed feedback, and we made revisions based on that feedback. Twelve parents replied saying that they did not have any edits and the map and tables reflected their experiences.

## Results

Participants included 22 parents and one former patient from 22 families. Most families had one parent attend the focus groups with two exceptions. There was one couple that attended together whose child had received exposure therapy, and another participant was a young adult who had received exposure therapy as a child. Responses to the RCADS and CGI-S-P indicated variability in severity of children’s anxiety and OCD symptoms at their worst. Participant demographics are shown in Table 1.

**Table 1:** Participant demographics (*N* = 23).^1^

Variable	ACE study participants (*N* = 19^1^) *M*(*SD*) or *N*(%)	IMPACT advisory group participants (*N* = 4) *M*(*SD*) or *N*(%)	All participants (*N* = 23) *M* (*SD*) or *N* (%)
RCADS-25 score—worst (*N* = 18)	27.61 (10.80)	n/a	n/a
CGI-S-P—worst (*N* = 18)	5.22 (1.00)	n/a	n/a
CGI-S-P—current (*N* = 18)	2.67 (1.03)	n/a	n/a
Parent age	45.05 (4.02)	40.25 (10.69)	44.22 (5.68)
Parent gender			
Female	17 (89.5%)	4 (100%)	21 (91.3%)
Male	2 (10.5%)	-	2 (8.7%)
Parent race			
Race not listed: “Hispanic”	1 (5.3%)	-	1 (4.3%)
White	15 (78.9%)	4 (100%)	19 (82.6%)
Prefer not to say	2 (10.5%)	-	2 (8.7%)
Missing	1 (5.3%)	-	1 (4.3%)
Parent ethnicity			
Hispanic or Latine	2 (10.5%)	-	2 (8.7%)
Not Hispanic or Latine	14 (73.7%)	4 (100%)	18 (78.3%)
Prefer not to say	2 (10.5%)	-	2 (8.7%)
Missing	1 (5.3%)	-	1 (4.3%)
Parent highest level of education			
High school	1 (5.3%)	-	1 (4.3%)
College graduate (2-year)	1 (5.3%)	-	1 (4.3%)
College graduate (4-year)	7 (36.8%)	1 (25%)	8 (34.8%)
Some college	2 (10.5%)	-	2 (8.7%)
Master’s degree or equivalent	7 (36.8%)	1 (25%)	8 (34.8%)
Doctoral degree or equivalent	1 (5.3%)	2 (50%)	3 (13.0%)
Household income			
35,000–49,000	1 (5.3%)	1 (25%)	2 (8.7%)
75,000–99,999	2 (10.5%)	-	2 (8.7%)
100,000–149,000	5 (26.3%)	-	5 (21.7%)
150,000–199,999	3 (15.8%)	1 (25%)	4 (17.4%)
200,000 and over	6 (31.6%)	2 (50%)	8 (34.8%)
Prefer not to say	2 (10.5%)	-	2 (8.7%)
Child age	12.94 (3.65)	13.33 (3.22)	13.00 (3.52)
Child gender			
Female	4 (22.2%)	2 (66.7%)	6 (28.6%)
Female and Non-binary	1 (5.6%)	-	1 (4.8%)
Male	13 (72.2%)	1 (33.3%)	14 (66.7%)
Child race			
Race not listed: “Hispanic”	1 (5.6%)	-	1 (4.8%)
White	15 (83.3%)	3 (100%)	18 (85.7%)
Prefer not to say	2 (11.1%)	-	2 (9.5%)
Child ethnicity			
Hispanic or Latine	2 (10.5%)	1 (33.3%)	3 (13.6%)
Not Hispanic or Latine	14 (73.7%)	2 (66.7%)	16 (72.7%)
Prefer not to say	1 (5.3%)	-	1 (4.5%)
Missing	2 (10.5%)	-	2 (9.1%)
Child health insurance type			
Employer-sponsored insurance	15 (78.9%)	3 (100%)	18 (81.8%)
Health insurance purchased *via* federal Health Insurance Marketplace	1 (5.3%)	-	1 (4.5%)
Both employer-sponsored and federal Health Insurance Marketplace	2 (10.5%)	-	2 (9.1%)
Missing	1 (5.3%)	-	1 (4.5%)

1 Although 22 families participated, one family had two parents who participated, and one IMPACT advisory group member was a young adult who was a former patient; thus, demographics are reported for 23 study participants and 21 children.

**Figure 1:**
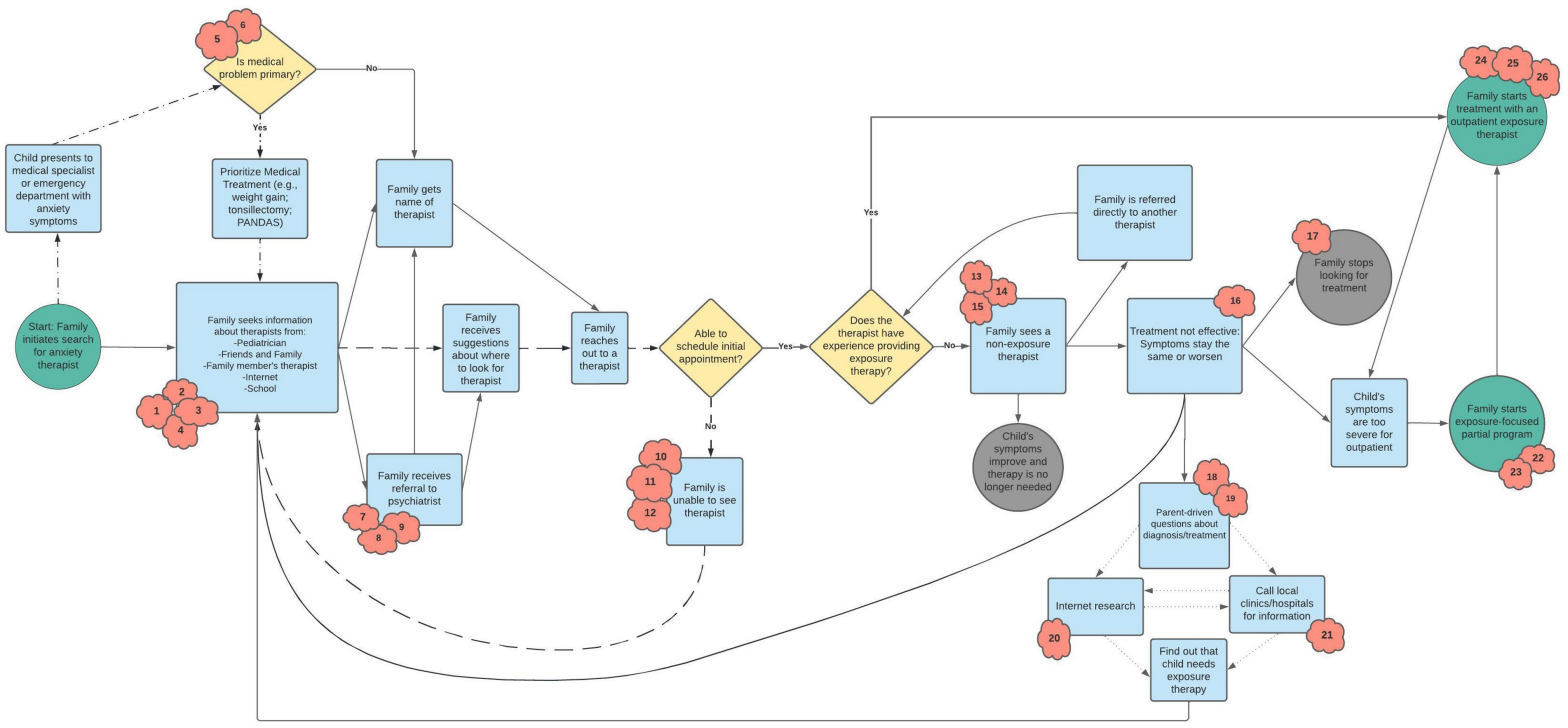
Final combined process map. Detailed descriptions of red clouds are provided in Table 2. Consistent with Kim and colleagues (31), each shape connotes a different type of step in the process: (a) Circles represent a process' start and end points; (b) Arrows between shapes depict the sequence in which events occur; (c) Rectangles indicate events that took place in the process; (d) Diamonds represent decision points in the process; and (e) Cloud shapes (referred to as "kapowies" during focus groups) represent uncertainties, gaps, bottlenecks, or inefficiencies. Loops are identified by different pattern lines: (a) the medical loop is represented by dashes and dots; (b) the search-outreach loop is represented by dashes; and (c) the parent research loop is represented by small dots.

The process map that emerged from our discussion with participants is shown in Figure 1. In Table 2, we describe the numbered “clouds” that appear in the process map, which represent gaps, bottlenecks, and uncertainties that occurred during the process of seeking treatment. This table also provides illustrative quotes from parents. Findings in Table 2 highlight challenges across each phase of seeking treatment—from initially looking for a therapist to starting therapy with a non-exposure therapist to eventually starting treatment with an exposure therapist. Personal connections and word-of-mouth were commonly cited as ways that parents found providers more quickly. Parents highlighted how structural barriers, such as waitlists, geographical location, and type of insurance accepted had an impact at multiple stages in the treatment-seeking process, including when they were first looking for the name of a therapist and when they found a therapist and initiated treatment. Parents frequently described “begging and pleading” (3703) to get into treatment, especially if they had been looking for a long time and their child’s symptoms were worsening. This resulted in emotional distress for parents, as well as initial willingness to stretch the family’s financial and other resources to initiate therapy (e.g., paying high out of pocket costs, traveling long distances). However, maintaining engagement in therapy that was expensive or geographically distant was often not sustainable or increased stress on the family system. In addition, when an initial course of treatment ended, parents cited difficulty re-engaging in treatment with the same or a new provider due to many of the same obstacles they faced when first seeking treatment.

**Table 2:** Description of process map “clouds”—Uncertainties, gaps, and bottlenecks.

Cloud number	Treatment phase	Description	Examples	Illustrative quotes
1	Seeking a therapist	Easier to find referrals if family member works in a medical or mental health field	Parent is a therapist and had coworkers who specialized in anxiety who could provide referral suggestions	“I got really lucky just that–since I am in the field, I knew the treatment I thought would be good for [anxiety] and I happened to know a couple providers who did that treatment, and one was willing to take [my son] on” (8608)
			Parent is a psychiatrist and was able to get expedited treatment for their child with another psychiatrist	
			Parent is a therapist and used connections to get in contact with other providers, but still had difficulty finding a provider with availability	“It was a very tough time for everybody in the household, it was tough trying to navigate the whole mental health system and knowing where to go. There wasn’t much out there” (4006)
2	Seeking a therapist	Reliance on word-of-mouth referrals	Parent had a close friend who works with children and adults with anxiety and OCD and recommended another provider	“We started asking friends for recommendations and actually when we did that, we realized that anxiety and other issues with kids were way more common than we thought. All my friends had recommendations for therapists and counselors…” (3511)
			Parents’ own therapist made a recommendation	
			Parent is a teacher and consulted coworkers (teachers, school psychologists), but ultimately found a therapist through a friend’s recommendation	
3	Seeking a therapist	Questions about etiology (e.g., PANDAS)	PANDAS was the primary diagnosis and required seeing one of few specialists in the country to learn about appropriate treatment options	“The pediatrician did do a workup because we were trying to rule out PANDAS because it seemed to come on so suddenly” (1008)
			Medical providers prioritized assessment for PANDAS (or other medical etiology) as a potential diagnosis which delayed access to psychosocial treatment	
			Child had repeated strep infections and tonsillectomy. There was an early discussion of PANDAS, but never any further testing. Family is still curious about PANDAS connection	
4	Seeking a therapist	Parent did not know to ask for exposure	Child completed a partial program for gastrointestinal symptoms that were secondary to anxiety, but exposure was not a part of the treatment	“The social worker…just wasn’t seeming to have an impact on the anxiety. We were still having all our same problems after a few sessions. [My son] liked to go and would talk through things, but it did not really have an impact on the anxiety behavior that was disrupting our household.” (1008)
			Child received psychosocial treatment through a partial program, but it did not include exposure	
			Families express confusion about terminology, including CBT	
5	Seeking a therapist	Mental health concerns may not be understood by medical providers	Underlying medical conditions (e.g., history of high fevers, infections, gastrointestinal symptoms, and migraines) led medical providers to focus on treatment of medical symptoms and largely ignore psychological symptoms	“If pediatricians listened more closely to parents—you aren’t there making things up, you are looking for help, for guidance for answers…I do not feel like I was heard, it was a very long journey to get [treatment]” (5105)
				“If the pediatricians were more helpful with mental health and saw it as part of your health period… we would have gotten help a lot sooner and it would not have gotten so severe.” (1505)
6	Seeking a therapist	Medical provider may lack knowledge about anxiety symptoms and treatment	Pediatricians were frequently consulted for input on symptoms, but pediatrician was not always able to identify/diagnosis anxiety disorders or OCD	“It would have saved us 3 years of unnecessary poking and prodding if we knew it was anxiety” (1509)
			Family spent two years seeing gastroenterologist for symptoms, and the question of anxiety was never raised	“I wish the pediatricians saw our son’s anxiety as a symptom [of PANDAS] and not as the disorder as a whole, because of that disconnect, nothing was ever looked into, and we were consistently dismissed.” (1505)
7	Seeking a therapist	Limited options for medication providers	Pediatrician initially prescribed medication but was not willing to manage it on an ongoing basis because doing so was beyond their expertise	“I had a little bit of difficulty with the pediatrician prescribing… he needed something formally written from the therapist about why he needed [medication]. It was hard getting [the therapist] to get the paperwork over to the pediatrician and I had to go through this every time, because you start off with a small amount that does not have a therapeutic effect” (2708)
			Therapist was not willing to see child if he was not on medication	
			Pediatrician would not continue to manage medication if child was not seeing a therapist consistently	
			Psychiatrist was initially consulted to suggest a medication dose, but it was managed in an ongoing capacity by the pediatrician	
8	Seeking a therapist	Limited coordination of care across providers resulted in parents having to retell their child’s history repeatedly in the process of looking for a therapist	Insurance did not allow psychiatry and psychology appointments to be billed in the same week	“I wish there was more connection between… a psychologist and psychiatrist, like they are working together. [It’s hard] trying to figure out what story you told who and always explaining to different people and going between the pediatrician, psychiatrist, and psychologist and they all have their own ideas, and they are all really busy.” (2708)
				“We really needed everybody [a care team]… it was on my husband and I to be the social workers and figure it out, and get him the help he needed… lots of trial and error and time lost” (1505)
9	Seeking a therapist	Assessment may provide diagnostic clarity	Completing a phone intake at a specialty anxiety clinic was the first time parents understood their child’s diagnosis and learned about exposure	“[When] calling the [hospital] main number, and trying to get into a clinical program, there was a waitlist and I was referred to a research assistant for [research study]…I did a phone intake for [research study] and learned–I wasn’t even aware that exposure therapy existed” (1008)
			Family started a program that did not end up being a good fit due to lack of proper symptom assessment beforehand	
10	Seeking a therapist	Structural barriers (e.g., cost, travel time, insurance) that affect the ability to find a therapist and schedule an initial appointment	Parents endorsed many barriers, including long waitlists, calls not being returned, therapists not accepting patients, limited appointment availability, high costs, and long travel time to appointments	“The hard thing is the wait times between everything” (1509)
				“We ended up paying out of pocket because of our high deductible” (1105)
			Parents expressed frustrations surrounding wait-times between partial programs and outpatient treatment. They discussed how symptoms get worse during wait-time, leading families to re-enter partial programs	“[Parent’s therapist] gave me three names and I did some research and made some phone calls and basically begged to get in…every single one I called said they did not have openings.” (3703)
			Parents frequently mentioned the need to “beg and plead” providers to see their child for treatment	“It’s frustrating when you are…a parent and your child is displaying severe symptoms: you want help and you do not know how to help…you want someone to tell you how to make things better”(S4)
11	Seeking a therapist	Emotional strain on parents	Trying to understand diagnosis and find an appropriate provider put a strain on parents’ relationship (“unrelenting stress”)	“It’s incredibly isolating. You feel like every other kid you see is happy and well-adjusted…And of course you logically know it’s not true, but it feels that way when your kid is hurting.” (9607)
				“When [my son] was really frustrated…he would say ‘you did this to me, this is your fault, you passed these genes on to me’” (3511)
12	Seeking a therapist	Child’s symptoms worsening	Ended up being referred to partial treatment because symptoms worsened so significantly while looking for outpatient providers	“I had to do a lot of begging and pleading [to get into treatment]” (3703)
				“[My son] did really well with [the partial program]…After a year, he backslid a bit so now we are doing outpatient [treatment]” (S1)
13	Family starts treatment with a non-exposure therapist	Therapist may assign “mini exposures” or assign at-home exposures only	Saw therapist doing “mini exposures” for a year but it wasn’t enough to address symptoms	“They would ask either my husband or I to do stuff and we did not understand exposure therapy…We were not educated enough until we got to [specialty clinic] to truly understand what it meant for the whole family to be a part of it.” (1505)
			Therapist gave child and family a book on OCD but did not provide psychoeducation that child had OCD or do exposures in session	
14	Family starts treatment with a non-exposure therapist	CBT therapists may not do exposure	Family found therapist through IOCDF by searching for ‘pediatric OCD help’ but therapist did not do exposure	“CBT therapists do not necessarily do exposure.” (4909)
				“[Calling a specialty clinic] was the first time I heard about exposure, [before that, I] only knew about CBT.” (1505)
				“[A barrier to treatment was] the therapist’s knowledge of OCD symptoms… I felt gypped. Dishing out $150 a week and it made me angry… [They] did CBT but only talked about it, [they] did not do anything.” (3703)
15	Family starts treatment with a non-exposure therapist	Diagnostic confusion and/or dismissal of symptoms by provider	Child’s therapist dismissed family and told them nothing more could be done for symptom improvement (even though they were not doing exposure)	“We got connected with a therapist that was local who said she specialized in pediatric OCD. We went to her for two years and she did nothing. My daughter would go and play with toys, not talk about anything, we would not do any activities, it got to the point where after two years, [the therapist] dismissed my daughter and told us that there was nothing that she could do that we were not doing already.” (S2)
			Child’s therapist told family child had GAD, but parent felt child’s symptoms did not align with GAD; they later learned child had OCD	
			Child’s anxiety was triggered by anaphylactic reaction. Family and medical providers at first believed child was having additional reactions, but then found out it was anxiety after child used EpiPen and visited the emergency room on 3 separate occasions	
16	Family starts treatment with a non-exposure therapist	Child is more reluctant to go to therapy after experiences with treatment not being effective	Took three tries to get child into partial program because he did not want to acknowledge his OCD	“Once [children] are older and in crisis, it is harder to get them to realize they need help” (1509)
17	Family stops search for therapist	Family discontinues search for treatment due to relatively limited impairment from symptoms or due to difficulty accessing ongoing treatment	Child’s symptoms were relatively better and waitlists were long, especially during COVID-19 pandemic	“[My son] just kinda seemed to get more in a groove again with school so we just stopped therapy and also because of limited availability there was no flexibility—it was just too challenging to have therapy” (3703)
18	Family dissatisfied with treatment	Parent-driven questions about OC-spectrum disorders (e.g., tics) and PANDAS	Parent’s googling led to questions about PANDAS due to sudden onset of symptoms	“I got a little bit sidetracked during the process with so many people mentioning it could be PANS or PANDAS and getting him tested for that, which was a whole other ball game” (2708)
			Child presented with tics first, then parent learned about connection between tics and OCD, leading to questions about OCD	
			Child had tics and migraines, prompting family to seek out neurological treatment and medication which later led to therapy for OCD	
			Overlap/misdiagnosis of tics versus OCD versus anxiety	
19	Family dissatisfied with treatment	Parents experience challenging emotional reactions	Parents gave attention to the child in need, and then worried that it would be hard on the other children in the family	“I was never really a big fan of mental health medications, now seeing the positive effect it has had on [my son], I feel differently about it…I sit in the way, just with my own hesitance to seek treatment and ignorance of what [my son] may be going through…When you hear there is a waitlist it’s brutal, your poor kid is suffering, you need to do something and you feel helpless.” (4006)
			Parent experienced guilt, shame, worry, hopelessness, frustration	“We questioned ourselves the whole way. Why aren’t we addressing it?” (1606)
				“The other barrier might be the shame in letting others in on the fact that your kid might have a mental health issue, which is shameful of myself to even say that because of how passionate I am myself [about mental health], but I did not want anyone to know” (3703)
20	Family dissatisfied with treatment	Online resources about anxiety, OCD, and exposure therapy	Parents found websites and podcasts which helped them to understand OCD	“I just started googling moral and harm OCD thoughts…Right in front of me was Natasha Daniels’ website…I thank her for everything, because right on her website [it explains what moral OCD is]. I cannot believe his therapist missed this” (2708)
			Even when parents did search online for exposure, did not find resources that they now know to be helpful	“When I googled and looked on Psychology Today it was not very easy…Now that I am all connected and listen to podcasts [I know about treatment delivery options]…but how did that not pop up when I was googling [exposure]?” (3703)
				“Now we have TikTok therapy and there is so much in the culture about all this therapy-speak, but I do not think exposure therapy is there yet.” (8606)
21	Family dissatisfied with treatment	Information about exposure received from speaking to staff at hospitals/clinics	Spoke with intake coordinator at specialty clinic and learned about exposure therapy	“Before [the psychologist] could even see us, she spent probably 2 hours on the phone with [us] explaining what was going to happen. She did not have time to see us right away, but she was the only person who called us back and was willing to spend the time with us to go through what this process [exposure] would look like.” (1606)
			Phone call with hospital staff led to recommendation for parents to contact specialty anxiety/OCD clinic	
22	Family starts exposure-focused partial program	Partial program is an extra step that involves more burden and does not guarantee a referral to an outpatient therapist	Multiple parents took leaves of absence from their jobs so they could get their child to a partial program on time every day	“I took a leave of absence during the program. There was no way [to do it otherwise]. The first day I came back in tears and had to take a leave of absence, it was awful” (2708)
23	Family starts exposure-focused partial program	Partial program provides support for parents and children and reduces feelings of isolation and guilt	Parent group for families whose children were in a partial program was helpful	“He felt very comfortable with everyone, the whole team, and there were other kids that had similar issues…He felt like he was at the right place…being able to help other kids his age and talk to them. He looked forward to it, every day. It helped him a lot.” (1502)
				“A friend…connected me with a mom who had a son with OCD. Her son had been at [a partial program for OCD]. She was our lifesaver. She is one of my best friends and I have only known her a year.” (3703)
24	Family starts treatment with outpatient exposure therapist	Changes to treatment due to COVID and transition to telehealth	Waitlists were longer and only options were telehealth during COVID-19 pandemic	“[My son] was doing pretty well. When COVID hit, everything was online, and he is not much of a talker, and it takes a while to get to know him…so he has not followed up [with treatment]” (5105)
			Telehealth requires parents to have a bigger role in delivering exposures	“This year everything is telehealth [due to COVID], which has made it incredibly easy [scheduling wise], but I’m not sure how effective it is.” (S2)
				“Then COVID hit, it went to telehealth and I became her exposure therapist…because this telehealth wasn’t working… [We were] begrudgingly getting zero out of it, so I ended up taking what I learned from sitting in on these sessions and soaking everything in” (1606)
25	Family starts treatment with outpatient exposure therapist	Structural barriers (e.g., cost, travel time, insurance) to maintaining access to treatment after a provider is identified and/or treatment is initiated	Family found a therapist, but sessions were $500 an hour with no insurance option	“Depending on certain insurance [plans], they only cover certain providers within their network, sometimes that can be super limiting, especially when there is already a limited number of providers…it’s hard when you are trying to budget things, [weekly visits] add up” (S4)
			Parents had to pull children out of school early for treatment, sit in waiting room with children’s siblings, and miss work	
			Multiple families cited waitlists ranging from a few weeks to 6 months long	
26	Family starts treatment with outpatient exposure therapist	When treatment ends, it can be challenging to find a new provider	Can be hard to find a new therapist after being discharged from partial and if they do, it may not be a sufficient dose / frequency of treatment.	“It was frustrating, I felt alone with no help after we completed [the partial program]” (8053)
			Not guaranteed to continue to be connected to provider as symptoms wax and wane	“After [research study], that was a big surprise to me…no one could help us get in to see another therapist…You feel like you made all this progress, but now what? You see this new therapist, and you are starting from scratch” (1606)

CBT, cognitive behavioral therapy; GAD, generalized anxiety disorder; IOCDF, International OCD foundation; OCD, obsessive–compulsive disorder; OC-spectrum, obsessive–compulsive spectrum; PANS, pediatric acute-onset neuropsychiatric syndrome; and PANDAS, pediatric autoimmune neuropsychiatric disorder association with streptococcal infections.

Given the timing of data collection for this study (2020–2022), some parents mentioned the impact of COVID-19 on treatment seeking. In particular, parents described mixed reactions to the emergence of telehealth as a primary mode for treatment delivery during the pandemic. Some parents reported that it improved access to treatment by increasing ease of scheduling and reducing transportation barriers. Other participants identified challenges related to higher reliance on parents to conduct exposures rather than having a therapist guide the child through exposures *in vivo*. Another telehealth-related challenge was difficulty building rapport *via* Zoom, especially for youth with social anxiety.

In addition to the barriers to treatment seeking highlighted in Table 2, the process map contains three “loops” or stuck points that emerged. These loops are described in Table 3. First, participants identified a “search-outreach loop,” in which they repeated the cycle of looking for therapists, contacting them, and being unable to schedule an appointment due to factors such as cost, waitlists, and travel time. Second, parents identified a “medical loop” that involved repeated visits to their pediatrician or other medical provider to receive input on ways to manage anxiety/OCD symptoms. This was particularly salient among parents whose children had symptoms that required treatment by medical providers, though many parents whose children did not have medical symptoms also endorsed frequent visits to the pediatrician to seek help related to anxiety symptoms. Several participants reported that their child’s pediatrician was a source of constant support and guidance through the treatment seeking process. However, other parents expressed frustration with pediatricians and medical specialists not having sufficient knowledge or resources to support them.

**Table 3:** Loops.

Loop pattern	Loop name	Loop description	Potential solutions
Dashes 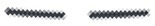	Search & Outreach Loop	• Repeated process of getting a name of a potential therapist and running into barriers to scheduling an appointment with them	• More resources at children’s schools (e.g., guidance counselors who know what exposure is, can teach parents skills, and provide referrals or exposure treatment) *(parent-generated solution)*
		• Requires substantial time from parents to seek services for their child (e.g., due to phone calls, timing of available appointments)	• More transparency and up-to-date information about referrals from insurance companies and professional organizations *(researcher-generated solution)*
			• Increase availability of lower intensity interventions (e.g., psychoeducation, assessment, phone consultation, single session interventions) *(researcher-generated solution)*
Alternating Dashes and Dots 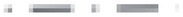	Medical Loop	• Presence of comorbid medical conditions OR medical symptoms (e.g., gastrointestinal distress, PANDAS, low weight due to anxiety/OCD symptoms) requires initial diagnosis and treatment by medical provider	• Dissemination of information and training for medical professionals, especially pediatricians, about anxiety/OCD assessment and exposure therapy *(parent-generated solution)*
		• Some children present to emergency room or medical provider for symptoms that can primarily be explained by anxiety/OCD, but may not be diagnosed accurately	• Integrated care teams that involve coordination with medical providers (specialists and pediatricians) and mental health providers (psychologists, psychiatrists, counselors) *(researcher-generated solution)*
		• Frequent visits to pediatricians for help with anxiety and associated medical symptoms	
Small Dots 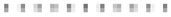	Parent research Loop	• After difficulty accessing appropriate treatment, parents did their own research into their child’s symptoms and possible treatments	• Dissemination of information about anxiety, OCD, and exposure therapy for parents *(parent-generated solution)*
		• Parents (and adolescents) looked up information online and called local clinics/hospitals for information	• Development of educational materials, such as podcasts and toolkits, geared toward parents to help recognize symptoms of anxiety and OCD *(parent-generated solution)*
		• Parents learned about exposure therapy, which led them back to looking for potential referrals	• Parent support groups, especially opportunities for families waiting for treatment to talk to families who have already done exposure *(parent-generated solution)*
			• Media promotion of exposure therapy (e.g., *via* television and radio commercials) *(researcher-generated solution)*
			• Psychoeducation about accommodation / importance of approach behaviors while waiting for treatment to give parents guidance on what to do *(researcher-generated solution)*

Finally, there was a “parent research loop” that involved parents doing their own research into anxiety and OCD treatment after not receiving clear guidance from medical providers and/or therapists. Some parents mentioned that they had also sought counseling for their child through school, but that it was not sufficient to address ongoing symptoms. In response to these failed attempts at seeking adequate treatment for anxiety and OCD, several parents described efforts to do online research and to make calls to local clinics and hospitals about what treatments might work for their child. They often noted confusion about their child’s diagnosis and the use of online research to gain a better understanding of their child’s symptoms [e.g., morality OCD, Pediatric Autoimmune Neuropsychiatric Disorders Associated with Streptococcal Infections (PANDAS)]. The role of diagnostic confusion was particularly pronounced for children with OCD caused by PANDAS. This is likely due to the relative rarity of PANDAS (41) and many providers’ lack of familiarity with this etiology. Parents differed in their experiences with PANDAS, where some said that assessment for PANDAS was a distraction, whereas other expressed frustration that an immune-related etiology was not consider by pediatricians. Many parents learned about diagnoses and exposure therapy through their own research, which allowed them to engage in a more directed search for their child’s treatment.

Finally, Table 4 describes the six overarching themes that emerged based on parents’ descriptions of the process of seeking treatment. These themes were derived from focus group notes and the process map itself, and include: (1) resources and terminology related to exposure therapy, (2) identifying diagnoses to guide treatment, (3) parent-related factors in treatment-seeking, (4) child-related factors that drive treatment seeking, (5) the role of medical providers, and (6) structural and social barriers to accessing care. In terms of resources and terminology related to exposure, parents described confusion about the variety of terms used to describe psychological treatments for anxiety disorders. After having received exposure therapy for his child, one parent said, “I keep hearing CBT but honestly, I do not even know what CBT means, what it stands for” (4006). Parents also talked about the importance of identifying diagnoses to guide their next steps for treatment. One participant noted, “I called one of my close friends who works with children and adults with OCD… [she] talked through it with me how OCD and tics are often overlapping and connected. That was really helpful” (8606). This parent went on to describe how understanding that OCD might be a part of the diagnostic picture led her to finding an appropriate provider. In the process leading up to finding treatment for their children, parents described many intense emotional reactions of their own that added to the challenge of seeking treatment. One parent said, “When you go through it [seeing your child in distress and not being able to help] every day, it’s torture” (1606). Child-related factors also affected the treatment seeking process. For instance, some parents described finding a therapist, but not being able to engage their child in treatment: “[The pediatrician] gave us a list of therapists to reach out to. We did try for about 3 months. [Child] would refuse to participate. He did not speak. We just sat there…” (5105). Medical providers, particularly primary care physicians and pediatricians, were frequently mentioned as the first places families went to get information to help their child. However, there was also acknowledgement that medical providers rarely receive specialized mental health training, particularly related to OCD. As stated by one parent, “There is not a single OCD CME [continuing medical education] out there, so if you are trying to target primary care providers as a way to get in, there is nothing out there” (3703). Finally, parents identified that there are many structural barriers related to accessing care, and how many of them had the social capital (e.g., colleagues in the mental health system) to help them access treatment. One parent described, “I think about other parents out there that aren’t educated [about mental health] or do not have resources or really good health insurance… there was no person stepping into our life helping us. That’s really scary for people who do not have all the resources to get the help” (1505). Many parents identified feeling lucky to have the resources available to seek treatment at a high financial or logistical cost even if it was a burden on their families.

**Table 4:** Emergent themes.

Theme name	Theme description
Resources/Terminology	• Varying terminology and acronyms such as exposure therapy, exposure and response prevention (ERP), CBT, and OCD cause confusion for parents
	• Parents did not know what kind of treatment to ask for to help anxiety and OCD symptoms
	• Parents benefitted from online resources about anxiety/OCD symptoms and treatment
Identifying diagnoses to guide treatment	• Challenging for parents to know what treatment to ask for if diagnosis is not clear
	• Receiving an appropriate diagnosis is a necessary precursor to finding the right treatment
	• Understanding the etiology of anxiety and OCD may be helpful in some cases and not in others
Parent-related factors in treatment seeking	• Parents’ emotional experiences while seeking treatment for their children are complex and include feelings such as isolation, hopelessness, guilt, and worry
	• Parents do not receive adequate support during the treatment-seeking process
	• Parents have limited guidance about the “right” way to respond to anxiety and OCD symptoms
Child-related factors that drive treatment seeking	• When a child’s symptoms are worsening or significantly impairing functioning, parents feel more urgency to find an appropriate provider, but it does not necessarily happen more quickly
	• Having multiple negative experiences with therapists may make youth more reluctant to seek treatment in the future
	• Finding a provider during transition times (e.g., after a partial program or when symptoms re-emerge after a period of improvement) is critical but challenging
Role of medical providers	• Medical providers may not receive specialized training in assessment and treatment of mental health concerns
	• Medical providers may offer medication, but there is a limited supply of providers who are able and willing to prescribe psychotropic medications to youth
	• Limited coordination of care across providers may require families to re-tell their story frequently
Structural and social barriers to accessing care	• Accessing care requires time, financial resources, “good” health insurance, and access to transportation to attend in-person appointments
	• Limited availability of appointment times requires parents to have flexible work schedules, or in some cases, required parents to take a leave of absence from work
	• Many families found therapists *via* personal connections (word-of-mouth) or because of their careers in medical or mental health fields

In addition, parents emphasized how helpful the focus groups themselves were, noting that the opportunity to talk to other parents was a valuable one. One parent said, “I would have paid a million dollars to talk to you all 3 years ago… this is priceless to be able to [talk to other parents]. Hearing your stories is validating because going through it you are trying to do the best thing, but you have no idea what that is” (1606). Another parent said, “I have not had the opportunity to talk to others about this ‘cause family and friends do not know about it or understand” (1105). Parents identified that having more support from friends, family, and/or medical providers during the treatment and treatment-seeking process would have improved their ability to support their child and had a positive impact on their own mental health. This is consistent with the finding that the process of seeking treatment brought up many negative emotions for parents, including isolation, guilt, and helplessness.

## Discussion

This study examined parents’ experiences accessing exposure therapy for their children. Parents participated in focus groups that used process mapping to guide conversations about their experiences accessing treatment. This approach allowed for parents and researchers to visualize the barriers and facilitators faced during this process and to identify stuck points to target in future implementation efforts. Results from this study indicate that parents seeking treatment for their children found themselves caught in “loops” (i.e., a “search and outreach loop,” a “medical loop,” and a “parent research loop”) in which they engaged in repeated process steps due to the emergence of barriers. In addition, parents reported feeling reliant on guidance from pediatricians and word-of-mouth recommendations from their personal networks to gain information about treatment options. Furthermore, parents reported feelings of isolation and guilt caused by the burden of finding treatment for their children with limited support.

### Targeting the three “loops” for future implementation efforts

The three “loops” identified in the process map represent the biggest stuck-points parents faced during treatment-seeking given that the barriers maintaining these loops persisted in the face of multiple attempts by parents to bypass them. Hence, these parent-identified loops highlight clear areas for future implementation efforts. The “search and outreach loop,” in which parents repeatedly received information about potential therapists but were unable to start treatment, was maintained through logistical barriers that have been identified in prior research [e.g., (27)]. These include parents’ lack of access to up-to-date information about available providers, long waitlists, high costs, and few providers who accept insurance. As a result, parents reported significant time and financial resources being dedicated to the search for a therapist for their child. To address the barriers maintaining the search and outreach loop, potential implementation efforts may focus on encouraging insurance companies to provide more up-to-date information about available providers, disseminating information about exposure to school-based providers, and increasing the availability of lower intensity interventions like phone consultations or single session interventions (42) that provide psychoeducation and tools for managing symptoms. The last column of Table 3 shows additional recommendations generated by participants and researchers for addressing this and other loops.

The second major loop was a “medical loop,” which involved frequently re-engaging with medical providers to manage anxiety-related medical symptoms. For some youth, the medical loop led to medical intervention (e.g., antibiotics to treat PANDAS; weight restoration) while for others it ultimately led to the discovery that symptoms were somatic (e.g., gastrointestinal distress caused by anxiety). Parents whose children required medical intervention noted that better coordination among medical and mental health providers [e.g., *via* integrated care teams; (43)] would have reduced their need to repeatedly explain their story and seek help from multiple providers. To address delays in obtaining an accurate diagnosis for medical symptoms that are secondary to anxiety, parents advocated for an increased focus on educating medical providers (primary care physicians and specialists) about anxiety, OCD, and exposure therapy. This may involve increased dissemination of information about identification of and recommended treatments for pediatric anxiety disorders and OCD, as well as additional training opportunities for medical providers. These results also further highlight parents’ interest in models that promote the integration of behavioral health services into primary care settings, which have shown promise for their effectiveness (44).

Finally, the “parent research loop,” in which parents and sometimes adolescents did their own research into symptoms and possible treatment options, was maintained by the limited available information about symptom presentations of and EBIs for anxiety and OCD. Parents reported feeling guilty about their lack of knowledge and inability to find a suitable provider, which motivated them to engage in their own research. Parents also emphasized the difficulty of navigating the mental health system; many reported that it took years to find an exposure therapist. One strategy to reduce time that parents spend in this loop is to disseminate information in a cohesive and user-centered manner such that parents can easily learn about EBIs and use this information to request their desired treatment by name. This is in line with calls for direct-to-consumer marketing as a strategy to increase provider use of EBIs for anxiety disorders (45). Parents also expressed a strong interest in family support groups in which families who have already accessed treatment advise families going through the process. Warmlines—confidential and often free peer-support lines staffed by volunteers (46)—may offer a personalized peer-guidance option for parents. Warmlines may be particularly helpful given that they may address both the “search and outreach cycle” and the “parent research loop” by providing emotional and logistical support during the treatment-seeking process.

### Implications of uncertainties experienced, system barriers, and equity considerations

#### Uncertainties experienced by parents throughout the process

An overarching emotion that arose repeatedly in focus groups was the presence of confusion throughout the treatment seeking process. Specifically, parents endorsed ongoing diagnostic uncertainty and confusion about what EBIs for anxiety and OCD entail, which in turn brought about feelings of guilt, hopelessness, and worry. A particularly consequential finding in this study was that multiple parents expressed confusion about what treatment to ask for due to the number of terms used to describe EBIs for anxiety and OCD (e.g., exposure therapy, ERP, and CBT). Other parents who felt confident about the meaning of relevant terms expressed frustration and uncertainty about how to ensure their child’s provider was really offering the treatment they advertised (e.g., providers delivering CBT but not doing exposure). These findings further highlight the need for increased dissemination about EBIs for anxiety and OCD to parents to increase demand, as well as increased training and consultation for therapy providers. It also underscores the importance of considering the end-user, or consumer, of EBIs when it comes to intervention development and implementation (47). Related to this, efforts to “rebrand” exposure should consider terminology that will be more intuitive and less confusing to parents and families (48).

#### System barriers to medical providers playing a supportive role

One theme that has relevance to all of the identified “loops” is the role of medical providers. One concern was that physicians do not have adequate training to diagnose or determine effective treatments for mental health disorders. Indeed, prior research suggests that pediatricians may not be well trained in recognizing mental health disorders, including OCD (5, 49). However, parents’ distress about mental health symptoms during primary care visits may increase provider recognition of mental health disorders (49). Although this highlights the importance of advocating for their children, some parents reported that their child’s anxiety or OCD symptoms were dismissed by medical providers. This is in line with past research suggesting that supportiveness or dismissiveness from professionals is a determinant to accessing treatment (27). At the same time, physicians face significant barriers to providing parents with mental health support. A systematic review found that pediatricians identified low confidence and knowledge about mental health, limited time, low reimbursement, and lack of resources as the biggest barriers to recognition of mental health problems in their youth patients (50). Furthermore, barriers to pediatricians making referrals to specialist services are similar to those faced by parents and include lack of providers and resources, waiting times, and insurance coverage (50). These findings indicate that even if pediatricians have the knowledge to identify mental health symptoms, healthcare system barriers prevent them from making referrals that parents find helpful. Ultimately, in addition to pediatricians’ understanding of mental health disorders, their perceptions of parents and their own access to resources (e.g., a referral database) impact their clinical decisions. Additional research is needed to assess physicians’ perspectives related to providing mental health assessment and referrals, which can guide the development of implementation strategies to address this area of need.

As noted above, implementing integrated behavioral health services in primary care physicians’ offices is one strategy to address barriers related to communication between physicians and therapists (43). For instance, integrated behavioral health providers (therapists) can provide consultation directly to primary care physicians about mental health assessment. In addition, this model supports consultation to patients *via* “warm handoffs” from physicians to behavioral health providers, as well as increased coordination of care between medical providers and behavioral health providers. The ability to receive brief behavioral health interventions in primary care physicians’ offices addresses barriers of finding an appropriate provider and reduces the number of times families must retell their story and start over with a new provider. In addition, by providing services within a clinic where the family is already accessing care, it is likely to reduce structural barriers such as treatment costs and transportation. Although there are some families for whom the brief care provided in primary care offices will not be sufficient, integrated behavioral health providers tend to be well positioned to provide appropriate community referrals.

#### Equity considerations in the treatment-seeking process

Study participants identified several equity considerations that warrant further consideration in efforts to improve access to care for youth. Parents’ concerns are consistent with the literature on disparities to accessing treatment, particularly among youth who are minoritized and underserved. For instance, previous research indicates that Black and Latinx youth are significantly less likely to receive needed mental health treatment compared to White youth (51–53). Race and ethnicity were not explicitly mentioned by parents during focus groups; however, inclusion criteria for this study required that families received exposure therapy. The relatively low rates of racial/ethnic minoritized participants in this study may in part reflect the fact that fewer racial/ethnic minoritized youth access effective therapy due to a range of socio-ecological factors (54, 55).

Sociodemographic variables, including poverty, have also been demonstrated to predict patterns of service use, with lower rates of adequate care for individuals living in high-poverty areas (56, 57). Consistent with Lu and colleagues’ (55) systematic review findings related to the contextual/structural and social/cultural levels of the Social Ecological model, several parents in this study specifically acknowledged that they had the resources to overcome common barriers to accessing mental health treatment. For instance, parents were able to overcome geographical barriers, such as where providers’ offices are located. Multiple parents discussed the need to go out of state for treatment, with at least two parents relocating for a period to access treatment for their children.

Parents identified additional facilitators they experienced, such as having financial or social resources to allow them to follow a path to accessing treatment. For instance, many parents noted that personal or professional connections with providers allowed them to get into treatment more quickly. This parent-reported dependence on their own networks and social capital further showcases how the gap between high-resource and low-resource families is maintained, given that higher income individuals are more likely to have greater health literacy and comfort navigating the medical system (58, 59). Thus, future research examining access to treatment should work to include the perspectives of parents from a range of cultural and socio-economic backgrounds that may not have the social or cultural capital to circumvent barriers. This can inform tailored approaches to addressing barriers to treatment access. For instance, community outreach programs for youth who are underserved and from racial/ethnic minoritized groups may improve social network support and improve word-of-mouth referrals to culturally responsive exposure therapists (55).

### Strengths, limitations, and next steps

Strengths of this study include its focus on obtaining data informed by parents’ perspectives and the novel application of the process mapping approach to obtain detailed information about barriers to accessing mental health treatment. A strength of process mapping itself is its ability to showcase the timing in which a barrier arises during a process and the steps that both precede and follow that barrier. Process mapping is complementary to “group model building” (GMB), a system dynamics-based method in which contributors develop a causal loop diagram that models problems and opportunities for improvement (22, 60). The identification of barriers through process mapping can help inform GMB and causal loop diagrams, which in turn will further improving tailoring of implementation strategies to match determinants (22). Process mapping may be a method particularly well-suited to presenting parents’ and patients’ perspectives, as it allows participants the opportunity to see their story represented visually and to provide feedback and clarifications in real-time.

In addition to these strengths, this study had multiple limitations. Recruitment was geographically restricted to families seeking treatment in Rhode Island and Massachusetts; thus, participants may have experienced barriers or facilitators to treatment access specific to New England. Additionally, we had some difficulty scheduling participants due to their work schedules. Although we offered a variety of times for focus groups, some parents were unable to predict work schedules in advance to commit to any meeting time. This is a major limitation given that the available participants had the flexibility in their schedules to attend the focus groups, and therefore, likely had similar resources that increased their access to treatment. This limitation was referenced by many participants themselves, who noted that they could not imagine the obstacles faced by parents who had less time and fewer resources. Consequently, this study may also be inherently skewed toward the perspectives of families with greater resources, as we recruited parents who had already accessed exposure therapy for their children. In particular, this sample had a preponderance of White, highly educated parents with relatively high incomes, which does not represent the larger population of people who may seek treatment for mental health concerns. Another limitation of this study is that it focused only on families who successfully received exposure therapy; future research is needed to understand the perspectives of families who have not been able to receive exposure therapy or other types of therapy. Furthermore, except for one former patient, participants in this study were parents. Additional input is needed from youth to understand their perspectives on accessing treatment, including how they perceive the role of their parents in the treatment-seeking and utilization process.

The results of this study, along with its strengths and limitations, highlight future opportunities for research to help tailor implementation strategies to improve access to mental health care. While this study focused specifically on access to exposure therapy for youth with anxiety and OCD, it is likely that process mapping can highlight stuck points requiring intervention for other disorders and their EBIs. Our findings point to several directions for future interventions such as the creation of a streamlined referral process and user-friendly database of available providers, and the development of parent support groups or Warmlines to help families navigate the mental health system. Additional interventions may focus on enhanced coordination of care between medical and mental health providers by testing education programs for pediatricians and by examining collaboration efforts between medical and mental health providers. Future research with parents who are actively in the treatment seeking process could also shed light on the perspectives of more families including those who may not have the resources to readily access treatment. In addition, future research might benefit from explicitly asking parents to identify the relative importance of each barrier to prioritize the barriers that most urgently need to be addressed.

### Conclusion

Using process mapping, this study identified determinants to accessing exposure therapy for parents of youth with anxiety and OCD. Findings highlight priority areas to improve access to care, including: (1) lack of clarity regarding diagnosis, treatment, and terminology related to anxiety disorders and OCD; (2) parents’ experience of repeatedly encountering the same barriers during their attempts to access treatment and resultant feelings of guilt, shame, and helplessness; (3) inequitable access to care that relies on parents’ use of their own personal connections, flexible work schedules, and high costs for care; and (4) overreliance on medical providers, and particularly pediatricians, to solve issues related to accessing mental health treatment for anxiety and OCD. Although we specifically inquired about treatment for anxiety and OCD, many of these barriers likely expand to other populations. Future work should identify how barriers to accessing care are similar or different for other presenting problems and underserved populations. Furthermore, in partnership with parents and other key community members, future research should develop dissemination and implementation strategies specifically focused on addressing these barriers to accessing care.

## Data availability statement

The data presented in this article are not publicly available due to the highly sensitive nature of focus group data and risk of identifying research participants. Requests to access the datasets should be directed to the first author, HEF, hannah_frank@brown.edu.

## Ethics statement

The studies involving human participants were reviewed and approved by Rhode Island Hospital Institutional Review Board. Written informed consent for participation was not required for this study in accordance with the national legislation and the institutional requirements.

## Author contributions

HEF, KGB, and BK contributed to the conception and design of the study. HEF, EO’C, JK, and GC participated in the collection of study data. HEF and GC analyzed the study data and wrote the initial draft of the manuscript. HEF, GC, BK, and JF wrote and edited sections of the manuscript. All authors contributed to the article and approved the submitted version.

## Funding

This work was support by the National Institute of Mental Health [T32MH019927]. The funding source did not have any direct involvement in the study design, data collection, analysis, or writing of this report.

## Conflict of interest

The authors declare that the research was conducted in the absence of any commercial or financial relationships that could be construed as a potential conflict of interest.

## Publisher’s note

All claims expressed in this article are solely those of the authors and do not necessarily represent those of their affiliated organizations, or those of the publisher, the editors and the reviewers. Any product that may be evaluated in this article, or claim that may be made by its manufacturer, is not guaranteed or endorsed by the publisher.
